# Partial Hepatectomy Induced Long Noncoding RNA Inhibits Hepatocyte Proliferation during Liver Regeneration

**DOI:** 10.1371/journal.pone.0132798

**Published:** 2015-07-24

**Authors:** Lulu Huang, Sagar S. Damle, Sheri Booten, Priyam Singh, Mahyar Sabripour, Jeff Hsu, Minji Jo, Melanie Katz, Andy Watt, Christopher E. Hart, Susan M. Freier, Brett P. Monia, Shuling Guo

**Affiliations:** Department of Antisense Drug Discovery, Isis Pharmaceuticals Inc., Carlsbad, CA, 92010, United States of America; UT MD Anderson Cancer Center, UNITED STATES

## Abstract

Liver regeneration after partial hepatectomy (PHx) is a complex and well-orchestrated biological process in which synchronized cell proliferation is induced in response to the loss of liver mass. To define long noncoding RNAs (lncRNAs) that participate in the regulation of liver regeneration, we performed microarray analysis and identified more than 400 lncRNAs exhibiting significantly altered expression. Of these, one lncRNA, *LncPHx2* (Long noncoding RNA induced by PHx 2), was highly upregulated during liver regeneration. Depletion of *LncPHx2* during liver regeneration using antisense oligonucleotides led to a transient increase in hepatocyte proliferation and more rapid liver regeneration. Gene expression analysis showed that *LncPHx2* depletion resulted in upregulation of mRNAs encoding proteins known to promote cell proliferation, including MCM components, DNA polymerases, histone proteins, and transcription factors. *LncPHx2* interacts with the mRNAs of MCM components, making it a candidate to regulate the expression of MCMs and other genes post-transcriptionally. Collectively, our data demonstrate that LncPHx2 is a key lncRNA that participates in a negative feedback loop modulating hepatocyte proliferation through RNA-RNA interactions.

## Introduction

Although long noncoding RNAs (lncRNAs) such as *H19* and *Xist* were first discovered decades ago, only in the last few years have the advances in transcriptome sequencing technologies led to the discovery of thousands of previously unannotated lncRNAs [1, 2]. A recent update by the GENCODE consortium annotated 9,277 lncRNA genes that result in 14,880 transcripts [3]. Currently, lncRNAs are defined by two features: 1) the transcript is longer than 200 nucleotides and 2) does not appear to have coding potential. The first criterion of length is arbitrary and based on the purification method. Essentially, these RNAs are defined by what they are not rather than their function [2]. LncRNAs are often expressed in a tissue- and developmental stage-specific manner [1, 4], and levels of many are altered in disease states [5, 6]. Some lncRNAs have been shown to be essential for life and development, such as *Xist* [7], *Fendrr* [8, 9], and *Braveheart* [10]; others play important roles during disease progression [5], such as *Malat1* [11–14] and *Pvt1* [15]. Initially, it was thought that the main function of lncRNAs is to regulate transcription through influencing the epigenetic status of the chromatin [16]. More recently, however, lncRNAs that regulate almost every step of gene expression, including RNA processing, RNA stability, and translation, have been identified [2]. Yet the functions of most lncRNAs are unknown.

Previous studies have implicated lncRNAs in the regulation of cell proliferation [17]. To further understand the roles of lncRNAs in cell proliferation, we analysed lncRNA expression in a mouse two-thirds partial hepatectomy (PHx) liver regeneration model [18]. Liver regeneration after PHx is a very complex and well-controlled process, and requires participation of all mature liver cell types with hepatocytes being the main players [19–23]. Immediately following surgery, growth factors and cytokines work together to induce mature hepatocytes to re-enter cell cycle, which in turn triggers cell proliferation of the other cell types in the liver. Within 72 hours, hepatocytes complete 1 to 2 rounds of synchronized proliferation, and liver mass and function is fully restored in approximately 10 days. Liver mass is precisely controlled, as there is no over growth of the liver in response to PHx. A cascade of robust transcription regulation triggered by cytokine and growth factor signalling regulates this well orchestrated biological process [22, 24]. We performed genome-wide gene expression profiling to identify lncRNA expression changes during liver regeneration after PHx. We found that approximately 400 lncRNAs were differentially expressed after PHx. Interestingly, one lncRNA, *LncPHx2*, whose expression is induced after PHx, was shown to negatively regulate hepatocyte proliferation through inhibition of the genes that promote cell growth.

## Materials and Methods

### Animal experiments

PHx was performed as described before [18]. In brief, male Balb/c mice (Charles River Laboratories), 7~9 weeks of age were under isofluorane anesthesia (2% in air restrainer for induction and 1–2% via nose cone for maintenance). Left literal lobe and median lobe of the liver were removed with two separate ligatures. For experiments involving antisense oligonucleotide (ASO) treatment, mice were injected subcutaneously with LncPHx2_ASOs, control ASO, or PBS as indicated in the main text. The DEN-induced mouse HCC model was previously described [25]. In brief, male C57BL/6 mice, 15 days of age, were injected intraperitoneal with 25 mg/kg diethylnitrosamine (DEN, Sigma). A pool of DEN-injected BL/6 mice was maintained for 8 months to allow tumor formation, and then treated subcutaneously with ASOs or control reagents for 3 months before sacrificing and data collection. Animals were euthanized by exsanguination under Isoflurane inhalation followed by cervical dislocation. All animal husbandry and procedures were approved by the Institutional Animal Care and Use Committee at Isis Pharmaceuticals.

### Microarrays, RNA sequencing, and data analysis

Genome-wide profiling of mRNA and lncRNA expression changes during liver regeneration were performed using the NCode Mouse Non-coding RNA Microarray (Invitrogen). Data were normalized for intensity dependent variance using the vsn package (Bioconductor). Differentially expressed genes were identified and clustered using the maSigPro (microarray Significant Profiles) R-package. A two-step regression were performed to first identify significantly differentially expressed genes (FDR = 0.05), and then to identify the conditions that show statistically significant differences (alfa = 0.05, regression step = two.ways.backward). Genes were further filtered by goodness of fit gene profiles against gene regression models (R-squared > = 0.6), then aggregated into 9 clusters using hclust [26, 27]. To identify *LncPHx2*-regulated genes, mouse liver RNAs were analysed using Illumina True-seq protocol. Reads were processed using STAR [28]. Differential gene expression calls were made using cuffdiff [29]. GSEA analysis was done with pre-ranked gene list by expression [30].

### Antisense oligonucleotides

Antisense oligonucleotides used in this study were chemically modified with phosphorothioate in the backbone and constrained ethyl (cET) modifications in the wings with a central 10-nucleotide deoxy gap (3-10-3 gapmer). Oligonucleotides were synthesized using an Applied Biosystems 380B automated DNA synthesizer (PerkinElmer Life and Analytical Sciences, Applied Biosystems) and purified as previously described [31, 32]. ASO sequences are as follow: control ASO, 3-10-3 cET gapmer, 5’- GGCTACTACGCCGTCA-3’; LncPHx2_ASO1, 3-10-3 cET gapmer, 5’-AACTTCAAGTAACAGG-3’; LncPHx2_ASO2, 3-10-3 cET gapmer, 5’-AGGCATAACTTCAAGT-3’.

### Cell culture and transfection

Mouse cell lines MHT [33], BNL.CL2 (ATCC) and Hepa1-6 (ATCC) were cultured in DMEM containing 1% L-glutamine, 10% fetal bovine serum, and 100 units/ml penicillin/streptomycin in 5% CO_2_ at 37°C. ASOs were added to the culture media 5–12 hours after seeding cells at the indicated concentrations. Cells were harvest 48 hours after ASO-treatment.

### Plasma chemistry analysis

Blood samples were collected by cardiac puncture at time of sacrifice. Plasma chemistry values were measured on the AU480 Clinical Chemistry Analyzer (Beckman Coulter).

### RNA analysis

Cultured cells were lysed and the total RNA was extracted with Qiagen RNeasy columns. Animal tissues were homogenized in guanidine isothiocyanate solution (Invitrogen) supplemented with 8% 2-mercaptoethanol (Sigma-Aldrich). Total RNA was prepared using the Qiagen RNeasy columns. Quantitative real-time PCR (qRT-PCR) was performed using an ABI step-one sequence detector. Primer probe sequences are as follow: LncPHx1, forward, 5’-TGGATTTGGAAGCTTTGAGTGA-3’, reverse, 5’-CGTCTTTTCTCGGTGCTTGAT-3’, probe, 5’-FAM-CAGACACATGTTCCTCTTCCTCCTGCTCAX-TAMRA-3’; LncPHx2, forward, 5’-TGTTGCAGTGTGGTCCAGAGA-3’, reverse, 5’- CTGCTTCTTCTTCAGCAATGGAT-3’, probe, 5’-FAM- AGCCAGCCTTTTTGCTGTGGATCCCX-TAMRA-3’; LncPHx3, forward, 5’- GCACAGCACACTCAGAATTACAAA-3’, reverse, 5’- CCGCCTTTAATCCTAGCACTTG-3’, probe, 5’-FAM- ATGTATCCCTGGCTGGCTTGTAACCCAX-TAMRA-3’; LncPHx4, forward,5’- ACGCACCTTCCCCTGTCTT-3’, reverse 5’- TCCGCCTTCTCCATTTTGTG-3’, probe 5’-FAM- TTTGCCCTGTGTCCTTCTGTCTCCTGTT-TAMRA-3’; LncPHx5, 5’-GGGCTCCTCATGTGTTCG-3’, reverse, 5’-GGAATGGCAGAACTTCAGGA-3’, probe, 5'-FAM- TGGAAGGC-BHQ-1-3’; LncPHx6, forward, 5’-TGCCTTTGGCATTCTTTGTATCT-3’, reverse, 5’- GCAGTGCTGGTCCTCTGTGA-3’, probe, 5'-FAM-CTGCGTTTCACAGCAGCAGCCATCTAG-IOWA-BLACK (w/ internal ZEN) -3’, Ccnyl1, forward, 5’-TCGCTCCTTAGCAGATGACAAC-3’, reverse, 5’-CTTGAAATGGCCTCTAGGTTCTGT-3’, probe, 5'-FAM- ACCTGAATTTTCTGTTTGCTCCTCTCAGCA-IOWA-BLACK (w/ internal ZEN) -3’, Gapdh, forward, 5’-GGCAAATTCAACGGCACAGT-3’, reverse, 5’- GGGTCTCGCTCCTGGAAGAT-3’, probe, 5’-FAM- AAGGCCGAGAATGGGAAGCTTGTCATCX-TAMRA-3’. Taqman assay for Ccne1 (Mm00432367_m1), Mcm3 (Mm00801872_m1), Aurkb (Mm01718146_g1), Ccnb1 (Mm03053893_gH), and Rbl1 (Mm01250721_m1) were purchased from life technologies.

### Histological analysis

Animal tissues were collected, fixed in 10% buffered formalin, and paraffin embedded. Immunohistochemical analyses were performed using a Ki67-specific antibody (Thermo RM-9106-S) following manufacturer’s protocols. BrdU (Life Technologies, 00–0103) labelling was detected using BrdU *in situ* detection kit (BD, 550803). Single molecule RNA *in situ* hybridization was done using QuantiGene ViewRNA Assays (Affymetrix) following manufacturer's instructions.

### RNA interactome analysis

RNA interactome analysis was done as previously described [34]. In brief, fifteen biotinylated antisense DNA probes targeting the *LncPHx2* RNA were designed using the online designer at http://www.singlemoleculefish.com. Probes were divided based on their postions into two pools, odd pool (odd numbered probes) and even pool (even numbered probes). Eight biotinylated antisense DNA probes targeting the LacZ mRNA were used as negative control. Cells were fixed with 1% glutaraldehyde, and lysed in lysis buffer (50 mM Tris, pH 7.0, 10 mM EDTA, 1% SDS, added just before use: dithithreitol (DTT), phenylmethylsulphonyl fluoride (PMSF), protease inhibitor and Superase-In). Lysates were sonicated using Bioruptor (Diagenode) until completely solubilized. Cell lysate were diluted three times using hybridization buffer (500 mM NaCl, 1% SDS, 100 mM Tris, pH 7.0, 10 mM EDTA, 15% formamide, added just before use: DTT, PMSF, protease inhibitor, and Superase-In). Pooled probes (100 pmol) were added (1ul to 1ml of undiluted lysate), and lysate were incubated end-to-end rotation at 37°C overnight. Biotinlated probes and its associated RNPs were then captured using streptavidin-magnetic C1 beads (100ul beads/100pmol probes) and magnets (invitrogen) after wash (2×SSC, 0.5% SDS, fresh PMSF added, 5 mins at 37°C, repeat 5 times). For RNA elution, beads were treated first using proteinase K (1mg/ml, Ambion. proteinase K buffer:100 mM NaCl, 10 mM Tris, pH 7.0, 1 mM EDTA, 0.5% SDS. 50°C, 45 min, followed by boiling for 10 min). RNA was then isolated using Trizol reagent and was subject to DNase treatment (TURBO DNase kit, Ambion). qPCR analysis of *LncPHx2* and Gapdh levels were performed to determine the efficiency and purity of the experiment. cDNA libraries for RNA-seq were made using SMARTer Universal Low Input RNA Kit (Clonetech). RNA sequencing was done on an Illumina Hi-seq sequencer (single-end, 100-bp read length, 8 million reads). Sequences of probes targeting *LncPHx2* are as follow: #1, 5’-ATGGAAACCAGAATTCGCGC-3’; #2, 5’-CGAGTAACAAACTGCCGCAG-3’; #3, 5’-AAAACCAACTCTTCACCAGG-3’; #4, 5’-CATGGAGAGACCAAACTGCT-3’; #5, 5’- TGAGCAAAGGGAAGCTGTCA-3’; #6, 5’- ACATAGTTCTAGCAGATGCT-3’; #7, 5’- AGGCCAGAAAATGTCCAGAC-3’; #8, 5’- AACACATCCCTTTATCTTCT-3’; #9, 5’- ATCCACAGCAAAAAGGCTGG-3’; #10, 5’-TCTTCAGCAATGGATGGTGA-3’; #11, 5’-TAGTGTCAGGTGTGTTTGAC-3’; #12, 5’-GTGGAGAAGGGTGAGAAGAC-3’; #13, 5’-TAGGTATTTTTCAGTTCTGT-3’; #14, 5’- AATGCTAAAAGCAGGGGATC-3’; #15, 5’-AGTTTAGAGAAGTATGCCAT-3’. Sequences of control probes targeting LacZ are as follow: #1, 5’-ATTAAGTTGGGTAACGCCAG-3’; #2, 5’-AATAATTCGCGTCTGGCCTT-3’; #3, 5’- ATCTTCCAGATAACTGCCGT-3’; #4, 5’-AACTGTTACCCGTAGGTAGT-3’; #5, 5’- ACCATTTTCAATCCGCACCT-3’; #6, 5’- TGGTTCGGATAATGCGAACA-3’; #7, 5’- ATTTGATCCAGCGATACAGC-3’. Enrichment peaks were identified as described [34] with the following modifications. Coverage was computed at each position in the genome and an enrichment score (EScore) was calculated by computing the minimum coverage, scaled by number of mapped reads, between independently selected pull-down probe sets. Instead of an input sample, the minimum coverage score was normalized to lacZ probe set coverage to select against non-specific pull-down peaks. Adjacent EScores were merged using bedtools, and a mean EScore was determined. The average log2(EScore) and standard deviation of log2 (EScore) were 0.285 and 0.410, respectively. These regions had a minimum log2 (EScore) of 2, which corresponded to an enrichment greater than 4 standard deviations above the mean. The top 500 EScore regions were used in a *de novo* motif search in MEME [35]. To ensure that input peak sequences were not limited by narrow peak boundaries, the minimum input length was fixed at 100 bp (i.e., 50 bp flanking the peak centre). Reverse motif search using MAST was performed against the gene transcripts differentially expressed upon treatment with LncPHx2_ASO1, using unchanged gene transcripts as control [36].

### Statistics

Tow-tailed independent Student’s t test was used for statistical analysis in this study.

## Results

### Genome-wide lncRNA expression profiling during mouse liver regeneration after 2/3 PHx

**Fig 1:**
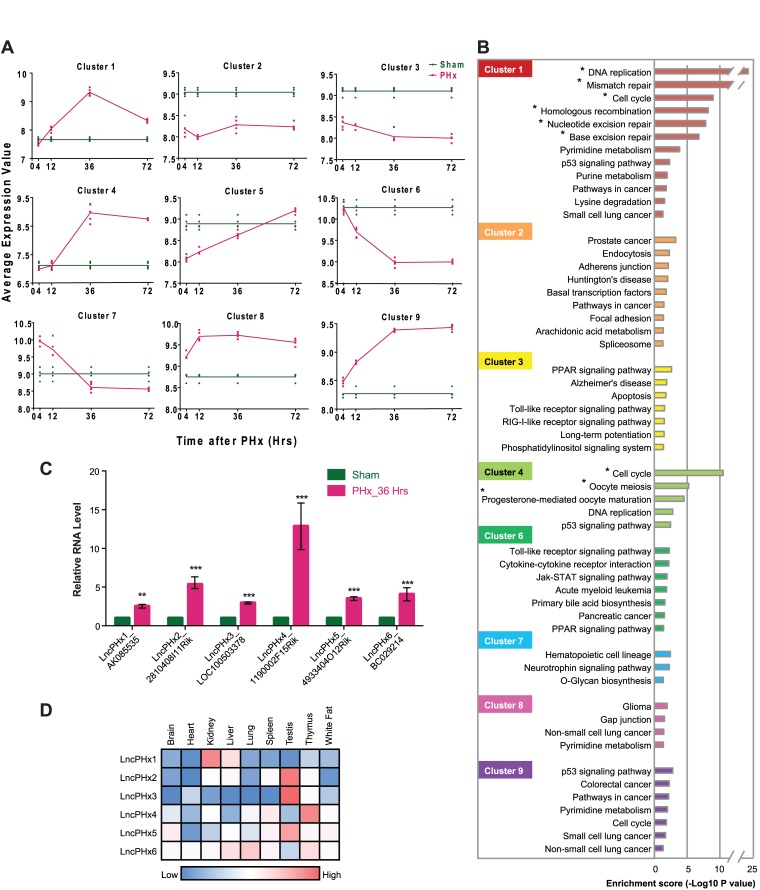
Gene expression profiling of mouse liver regeneration after PHx. (A) Differentially expressed mRNAs and putative lncRNAs were clustered based on their expression pattern during liver regeneration after PHx. Normalized average probe intensity was plotted over the time course of liver regeneration. Cluster 1 contains 401 mRNA and 30 lncRNA transcripts. Cluster 2 contains 471 mRNA and 91 lncRNA transcripts. Cluster 3 contains 610 mRNA and 110 lncRNA transcripts. Cluster 4 contains 385 mRNA and 46 lncRNA transcripts. Cluster 5 contains 146 mRNA and 17 lncRNA transcripts. Cluster 6 contains 410 mRNA and 73 lncRNA transcripts. Cluster 7 contains 254 mRNA and 37 lncRNA transcripts. Cluster 8 contains 330 mRNA and 17 lncRNA transcripts. Cluster 9 contains 646 mRNA and 44 lncRNA transcripts. Sham: liver RNAs of mice subjected to sham surgery. PHx: liver RNAs of mice subjected to PHx surgery. n = 5 for each time point. (B) Pathway analysis of the eight gene clusters using David KEGG pathway tools. Cluster 5 has no enriched pathway (not shown). * Pathway with FDR<0.05. (C) qPCR analysis of the levels of lncRNA transcripts. The mRNA level of the housekeeping gene *Gapdh* and total RNA amount determined by Ribogreen staining (Life Technology) were used as controls. The RNA levels in livers of mice subjected to sham surgery were set as 1. n = 5. Statistical analysis was performed using the Student's t test. * p<0.05; **p < 0.01; ***p < 0.001. (D) LncRNA expression in nine mouse tissues collected from Encode RNA-seq database. FPKM of lncRNA expression in each tissue was plotted against the mean value of each row. Red indicates higher expression. Blue indicates lower expression.

To identify lncRNAs that regulate cell proliferation during liver regeneration, we analysed lncRNA expression profiles in mouse liver tissue collected at 4, 12, 36, and 72 hours after PHx as synchronized hepatocyte proliferation occurs during this time window [37]. Analysis using the NCode Mouse Non-coding RNA Microarray (Invitrogen) revealed that 3653 mRNAs and 465 putative lncRNAs were differentially expressed compared to transcripts isolated from livers of control sham operated mice. Differentially expressed genes were categorized into nine clusters based on their expression patterns after PHx (Fig 1A and S1 Table). Clusters 1, 4, 8, and 9 contain genes that were significantly upregulated after PHx. Clusters 2, 3, and 6 contain genes that were significantly downregulated. Clusters 5 and 7 contain genes that were down- (cluster 5) or up- (cluster 7) regulated immediately after PHx and then the reverse at later time points. KEGG pathway analysis was performed on the coding genes in each cluster. We found that clusters 1and 4 were significantly enriched in genes regulating cell-cycle and cell proliferation (Fig 1B). We hypothesized that the lncRNAs within these two clusters might also affect cell-cycle and cell proliferation and, therefore, focused on the lncRNAs within these two clusters.

We manually curated putative lncRNA transcripts in clusters 1and 4 by using the UCSC genome browser to look for supporting evidence of mouse expressed sequence tags (ESTs) [38]. We also evaluated the coding potential of these putative lncRNAs using PhyloCSF [39]. qPCR primer/probe sets were designed to amplify the ten most upregulated transcripts that appeared to be lncRNAs based on supporting ESTs and lack of coding potential. Six of the ten lncRNAs were confirmed to be significantly upregulated during liver regeneration (Fig 1C). We named these 6 lncRNAs *lncRNA induced by PHx 1–6* (*LncPHx1-6*) (Fig 1C). Analysis of data obtained on nine mouse tissues available in the Encode RNA-seq database [40] showed that most of these lncRNAs are expressed in tissue-specific manners, with low to median expression in normal mouse liver (Fig 1D).

To study the functions of these lncRNAs, we designed antisense oligonucleotides (ASOs) to deplete them in cell culture and in vivo. We found that one lncRNA, *LncPHx2*, regulates hepatocyte proliferation in liver regeneration after PHx.

### *LncPHx2* is highly induced during liver regeneration

**Fig 2:**
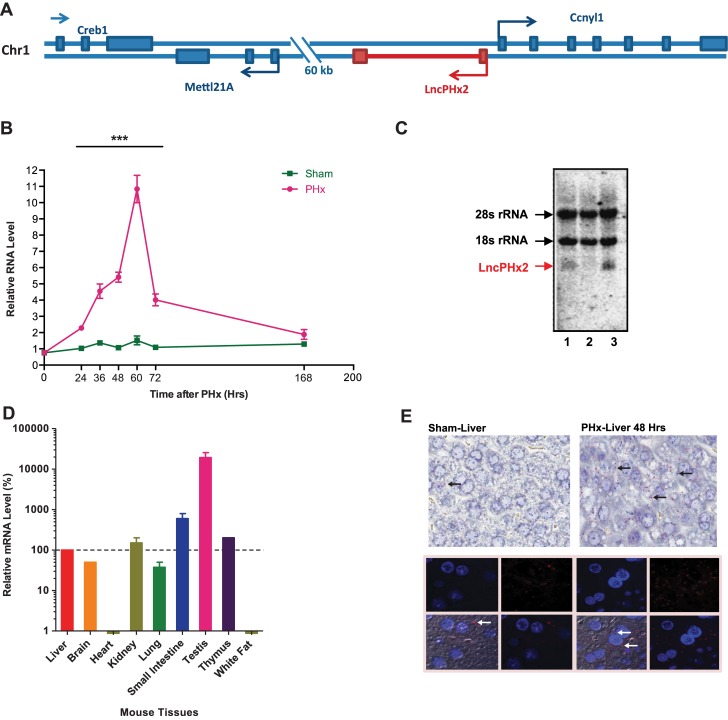
Characterization of *LncPHx2*. (A) *LncPHx2* gene structure and genomic location. (B) qPCR analysis of *LncPHx2* expression after PHx. The RNA levels in the livers at time 0 after sham surgery were set as 1. Data were quantified and statistically analysed as in Fig 1C. (C) Northern blot of *LncPHx2* in mouse livers. Lane 1, RNA from mouse liver treated with PBS. Lane2, RNA from mouse liver treated with ASO specifically targeting *LncPHx2*. Lane 3, RNA from mouse liver collected at 48 hours after PHx surgery. (D) qPCR analysis of *LncPHx2* levels in nine mouse tissues. *LncPHx2* level was normalized to total RNA amount measured by Ribogreen (Life Technologies). *LncPHx2* level in mouse liver was set as 1. (E) *LncPHx2* single molecule RNA *in situ* hybridization in livers of mice subjected to sham or PHx surgery. Upper panels: Light microscopy images. *In situ* signal of *LncPHx2* is red, as indicated by arrows. Liver sections were counter-stained by H&E. Lower panels: Fluorescent microscopy images. *In situ* signal of *LncPHx2* is red, as indicated by arrows. The nucleus was stained by DAPI.

*LncPHx2* (*2810408I11Rik*) from cluster 1, located on chromosome 1, is in proximity (553 bp upstream) in the antisense direction to a coding gene with unknown function, Cyclin Y like 1 (*Ccnyl1*) (Fig 2A). *LncPHx2* is highly upregulated during the hepatocyte proliferation stage of liver regeneration, between 24 and 72 hours after PHx, with peak expression observed at 60 hours after PHx (Fig 2B). Its neighbour gene *Ccnyl1* was slightly upregulated during liver regeneration (categorized in cluster 8) (S1 Fig). Three isoforms have been reported for the *LncPHx2* locus in Ensembl Genome Browser (S1 Fig); but only the 713-bp transcript (AK013046) could be cloned and detected by qPCR and by northern blot from mouse liver extracts (Fig 2B and 2C). *LncPHx2* was expressed in many mouse tissues with the highest expression detected in mouse testis (Fig 2D), confirming the Encode mouse tissue expression data (Fig 1D). Single molecule RNA i*n situ* hybridization showed that *LncPHx2* transcripts were localized mainly in the cytoplasm of hepatocytes in mouse liver (Fig 2E). *LncPHx2* RNA was also detected mainly in the cytoplasmic rather than the nuclear fraction in the cultured hepatocyte cell lines MHT and BNL.CL2 (S1 Fig and data not shown).

### *LncPHx2* regulates hepatocyte proliferation during liver regeneration after PHx

**Fig 3:**
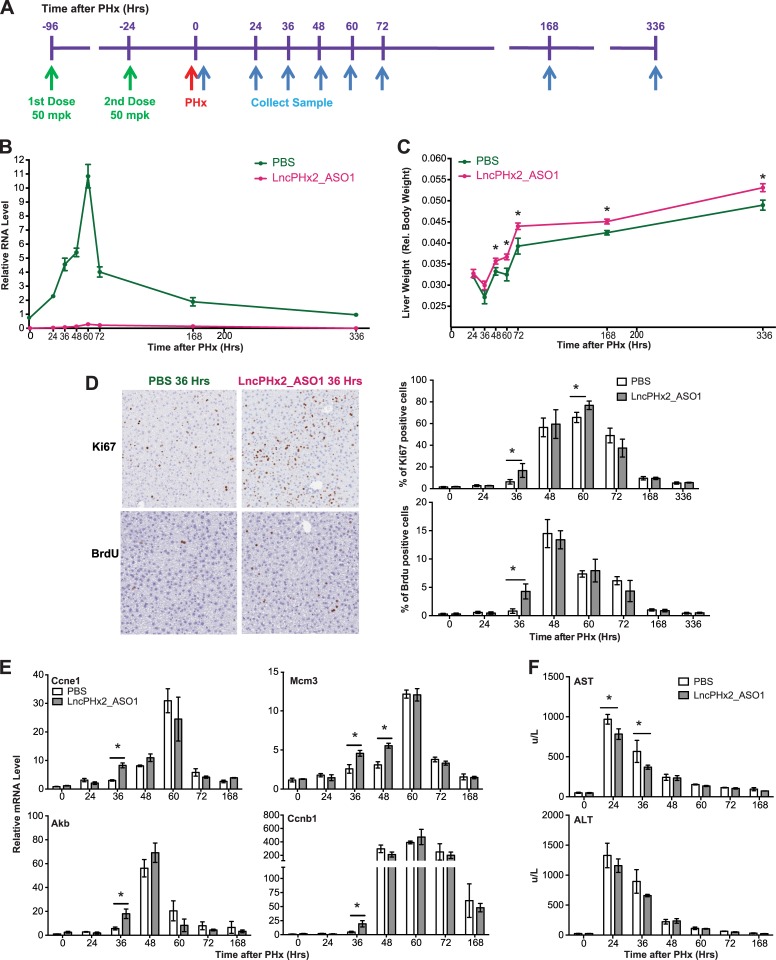
Depletion of *LncPHx2* promotes hepatocyte proliferation in liver regeneration. (A) Experimental procedure. Mice were given two doses of PBS or LncPHx2_ASO1 at 50 mg/kg (mpk) (indicated by green arrows) before PHx (indicated by red arrow). Animals were sacrificed at eight time points after PHx (indicated by blue arrows). n = 3 for 0, 24, 36, 48, 60, 72, and 168 hour time points. n = 4 for 336 hour time point. (B) qPCR analysis of *LncPHx2* in livers of mice treated with LncPHx2_ASO1 or PBS. The RNA levels in the PBS-treated livers at time 0 after PHx were set as 1. Data were quantified and statistically analysed as in Fig 1C. (C) Liver to body weight ratios measured at indicated time points after PHx. (D) Left panel: Representative images of Ki67 staining and BrdU labelling of livers from mice treated with LncPHx2_ASO1 or PBS at 36 hours after PHx. Right panel: Histogram and statistics of Ki67 staining and BrdU labelling of mouse livers after PHx at indicated time points after PHx. (E) qPCR analysis of cell-cycle marker gene expression in mouse livers at indicated time points after PHx. mRNA levels in the PBS-treated livers at time 0 after PHx were set as 1. Data were quantified and statistically analysed as in Fig 1C. (F) Liver aspartate transaminase (AST) levels (top panel) and alanine transaminase (ALT) levels (bottom panel) in mouse plasma measured at indicated time points after PHx.

To characterize the function of *LncPHx2*, we developed two ASOs that efficiently target *LncPHx2* for degradation by RNase H-mediated mechanism [31, 32](S2 Fig). We treated mice subcutaneously with LncPHx2_ASOs, and then performed PHx surgery as shown in Fig 3A. LncPHx2_ASO1 treatment completely blocked the upregulation of *LncPHx2* during the liver regeneration process (Fig 3B). Interestingly, mice treated with LncPHx2_ASO1 had more rapid liver weight recovery compared to PBS-treated mice (Fig 3C). The effect on liver weight was significant at 48 hours after PHx, and livers in LncPHx2_ASO1-treated mice remained heavier 15 days after PHx (Fig 3C). The body weights of LncPHx2_ASO1-treated mice were not significantly different from PBS_treated mice (data not shown). The same phenotype was observed with two different ASOs targeting *LncPHx2*, but not with scrambled control ASO (S3 Fig). More proliferating cells were observed at 36 and 60 hours after PHx in LncPHx2_ASO1-treated mice than in controls, as demonstrated by higher percentages of Ki67 staining and BrdU labelling (Fig 3D). No measurable differences in cell proliferation between LncPHx2_ASO1-treated and PBS-treated mice were observed at later time points (Fig 3D). qPCR analyses showed elevated expression of cell-cycle marker genes in LncPHx2_ASO1-treated mice including genes encoding Cyclin E1 (*Ccne1*), Cyclin B1 (*Ccnb1*), and Aurora Kinase B (*Aurkb*) at 36 hours after PHx, and Mini-chromosome maintenance complex component 3 (*Mcm3*) at both 36 and 48 hours after PHx (Fig 3E). *Ccnyl1* mRNA levels did not change upon *LncPHx2*-depletion in either regenerating livers or in livers subjected to sham surgery (data not shown). In addition, we observed a faster recovery of liver aspartate transaminase (AST) levels in plasma of LncPHx2_ASO1-treated mice than in controls, suggesting that suppression of *LncPHx2* resulted in a more rapid recovery of liver function (Fig 3F). As inhibition of *LncPHx2* expression led to increased hepatocyte proliferation after partial hepatectomy, *LncPHx2* appears to function as a negative regulator of cell proliferation during liver regeneration.

### *LncPHx2* suppresses the upregulation of positive cell-cycle regulators during liver regeneration

**Fig 4:**
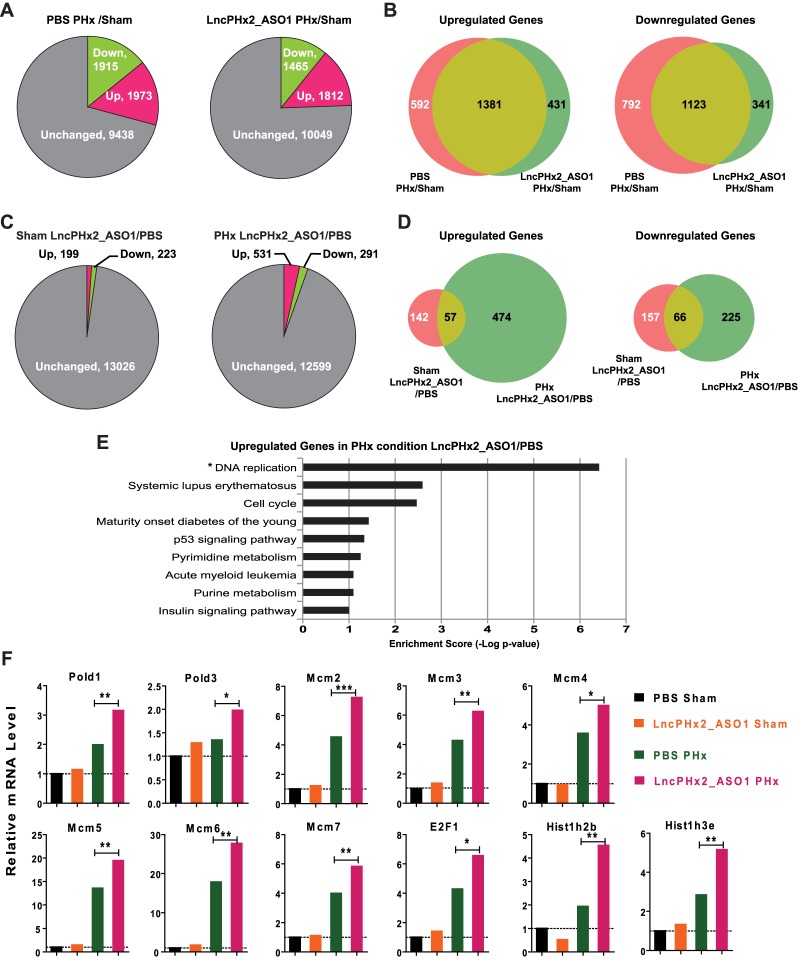
Genome-wide gene expression profiling of *LncPHx2* regulated genes. RNA-seq analysis of liver RNAs from mice treated with PBS or LncPHx2_ASO1 and subjected to Sham or PHx surgery. n = 3 for each group. (A) Pie charts of differentially expressed genes at 48 hours after PHx surgery compared to sham surgery in PBS-treated mice (left panel) and in LncPHx2_ASO1-treated mice (right panel). (B) Venn diagram comparing differentially expressed genes at 48 hours after PHx in PBS- and LncPHx2_ASO1-treated mice. Left panel: Upregulated genes. Right panel: Downregulated genes. (C) Pie charts of differentially expressed genes in LncPHx2_ASO1-treated mice compared to PBS-treated mice at 48 hours after sham (left panel) and PHx surgery (right panel). (D) Venn diagrams of differentially expressed genes in LncPHx2_ASO1-treated mouse livers under either sham or PHx conditions compared to PBS-treated mouse livers under the same conditions. Left panel: Upregulated genes. Right panel: Downregulated genes. (E) KEGG pathway analysis of genes upregulated in regenerating livers from LncPHx2_ASO1-treated mice compared to PBS-treated mice at 48 hours after PHx. * Pathway with FDR<0.05. (F) RNA-seq results of representative genes in each treatment condition indicated. mRNA levels in PBS-treated mice with sham surgery were set as 1. Ratios and statistics were done using cuffdiff. * p<0.01; **p < 0.001; ***p < 0.0001

To investigate the mechanism that underlies the function of *LncPHx2* in regulation of hepatocyte proliferation, we performed RNA-seq analysis on livers of mice subjected to PHx or to sham surgery after treatment with LncPHx2_ASO1 or with PBS. Gene expression profiling was performed at 48 hours post-surgery since it was at this time point that we first observed liver weight differences between ASO-treated and PBS-treated mice. In PBS-treated mice, 1,973 genes (14.8% of 13,326 expressed genes) were significantly upregulated, and 1,915 genes (14.3%) were significantly downregulated at 48 hours after PHx compared to gene expression in PBS-treated mice subjected to sham surgery (Fig 4A left panel and S2 Table). In LncPHx2_ASO1-treated mice, 1,812 genes (13.5%) were significantly upregulated, and 1,465 genes (11.0%) were significantly downregulated at 48 hours after PHx compared to mice treated with ASO and subjected to sham surgery (Fig 4A right panel and S2 Table). The overlap between the two groups included 1,381 upregulated genes and 1,123 downregulated genes (Fig 4B and S2 Table). Interestingly, there were nearly equal numbers of up- and downregulated genes in the PBS-treated mouse livers at 48 hours after PHx (14.8% upregulated genes vs. 14.3% downregulated genes), whereas there were more genes upregulated than downregulated in the LncPHx2_ASO1-treated mouse livers (13.5% vs. 11.0%).

When the data from LncPHx2_ASO1-treated mice subjected to sham surgery were compared to data from PBS-treated mice subjected to sham surgery, we found that 199 genes were significantly upregulated, and 223 genes were significantly downregulated (Fig 4C left panel, and S2 Table). These are likely the genes regulated by *LncPHx2* in normal livers. When data from animals subjected to PHx with LncPHx2_ASO1 treatment were compared to those treated with PBS, we found that 531 genes were significantly upregulated, and 291 genes were significantly downregulated (Fig 4C right panel, and S2 Table). These are likely the genes regulated by *LncPHx2* during liver regeneration. There were about equal numbers of up- (199) and downregulated (223) genes in sham liver in LncPHx2_ASO1-treated mice, whereas there were 1.8-fold more upregulated genes (531) than downregulated genes (291) in regenerating liver upon *LncPHx2* depletion (Fig 4C). The overlap between the two groups included 57 upregulated genes and 66 downregulated genes (Fig 4D and S2 Table). KEGG pathway analysis was performed to identify pathway enrichment in these differentially expressed gene subsets. We found that genes promoting cell proliferation were enriched in the genes upregulated upon *LncPHx2* depletion in regenerating livers (Fig 4E), but in livers of mice treated with ASO and subjected to sham surgery, no enrichment of these genes was found (data not shown). Genes that appear to be regulated by *LncPHx2* include those that encode DNA polymerases (*Pold1* and *Pold3*), mini-chromosome maintenance complex components (*Mcm2*, *3*, *4*, *5*, *6*, and *7*), histone proteins (such as *Hist1h3e* and *Hist1h4b*), and transcription factors that are involved in cell-cycle regulation (such as *E2F1*) (Fig 4F). The genes regulated are consistent with the increased cell proliferation phenotype we observed in *LncPHx2*-depleted regenerating livers. Moreover, the levels of these genes are not changed in *LncPHx2*-depleted mouse livers subjected to sham operation (Fig 4F), in which no increased cell proliferation was observed (data not shown). These results suggest that *LncPHx2* negatively regulates cell proliferation specifically in regenerating livers in response to PHx through modulating the expression of genes that promote cell proliferation.

### Gene expression signature of *LncPHx2*-depleted regenerating livers is similar to that of HCC tumours

To further investigate what pathways are regulated by *LncPHx2*, we employed Gene Set Enrichment Analysis (GSEA) to search for enrichment across the Molecular Signatures Database (MSigDB), after ranking genes according to differential expression. We found that genes involved in cell proliferation, such as those involved in meiosis and DNA synthesis were positively associated with the *LncPHx2*-depleted regenerating liver gene set (S4 Fig). Interestingly we also found that genes downregulated in cancers, were also downregulated in regenerating livers depleted of *LncPHx2* (S5 Fig). Comparison with the oncogenic signature datasets in MSigDB revealed that the genes upregulated by *LncPHx2*-depletion in regenerating liver is also upregulated when tumour suppressor retinoblastoma protein (RB), retinoblastoma-like 1 (RBL1), and breast cancer 1 (BRCA1) were depleted (S5 Fig). These correlations suggest that *LncPHx2* might function as a tumour suppressor.

To test this hypothesis, we employed a diethylnitrosamine (DEN)-induced mouse hepatocellular carcinoma (HCC) model, since genes downregulated in DEN-induced HCC were among the top negatively associated gene datasets to the *LncPHx2*-depleted regenerating liver gene set (S5 Fig). In addition, we found that *LncPHx2* expression was moderately upregulated in tumours from mice with DEN-induced HCC compared to levels in adjacent tissue (S5 Fig). DEN-treated mice were injected with PBS, control ASO or LncPHx2_ASO1 subcutaneously (S5 Fig). *LncPHx2* expression in tumour cells was significantly reduced in mice treated with LncPHx2_ASO1 compared to levels in tumour cells from control mice (S5 Fig). The tumour volumes, however, were indistinguishable between those from LncPHx2_ASO1-treated mice and PBS- and control ASO-treated mice (S5 Fig). These results indicate that the depletion of *LncPHx2* alone is not sufficient to augment tumour progression in the DEN-induced HCC mouse model.

### *LncPHx2* interacts with many mRNAs including several encoding proteins involved in cell proliferation

**Fig 5:**
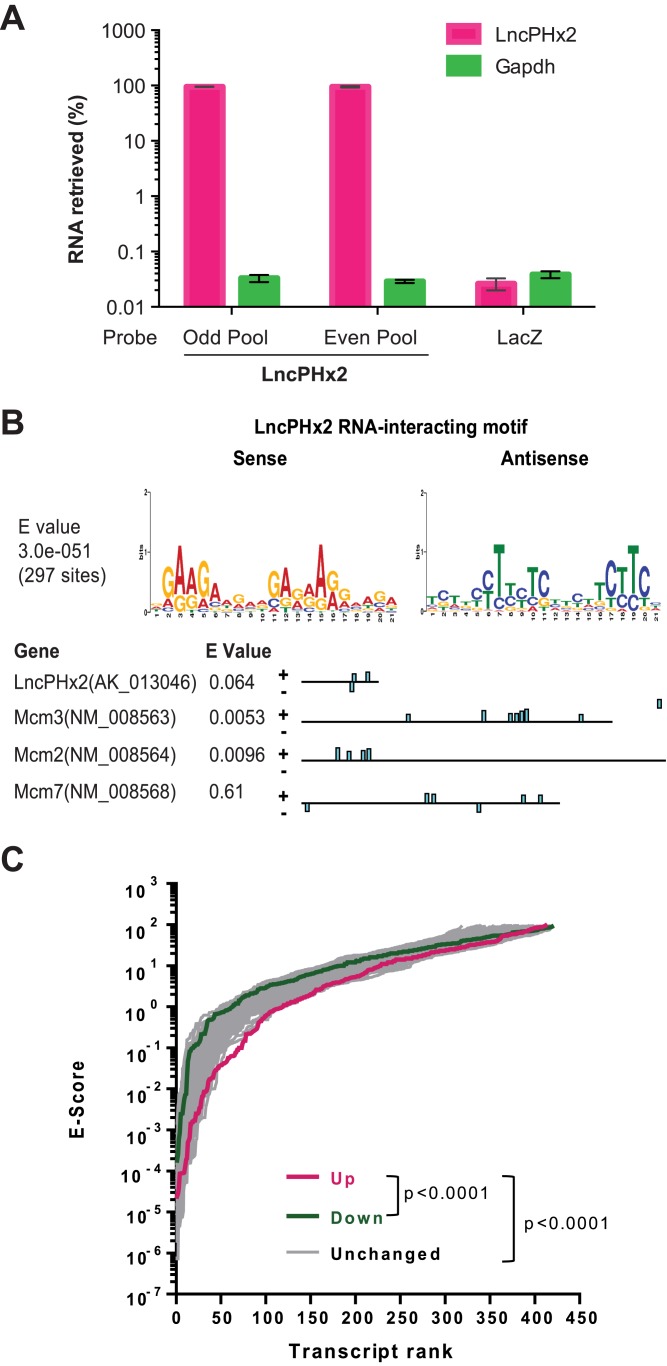
Identify *LncPHx2* RNA-interactome. (A) qPCR analysis of *LncPHx2* recovery in RNA samples from *LncPHx2* RNA-interactome experiment. Odd pool: pool of odd numbered probes bind to *LncPHx2* RNA. Even pool: pool of even numbered probes bind to *LncPHx2* RNA. LacZ: control probes bind to LacZ mRNA. (B) Upper panel: *LncPHx2* RNA-interacting motif identified from 415 *LncPHx2* interacting sites using MEME de novo motif search tool. Lower panel: *LncPHx2* RNA-interacting motif in *LncPHx2*, *Mcm2*, *Mcm3* and *Mcm7* transcripts. (C) *LncPHx2* RNA-interacting motif enrichment in differentially expressed genes in regenerating liver upon *LncPHx2*-depletion. Motif-search for the *LncPHx2* RNA-interacting motif was performed on 300 upregulated, 291 downregulated and 100 sets of 300 randomly sampled unchanged gene transcript sequences in *LncPHx2*-depleted regenerating liver using MAST with default parameters (e-value cutoff 100, maximum p-value for motif match = 0.0001). Student’s t-test was performed on log-transformed e-scores from the 3 groups.

Cytoplasmic lncRNAs have been shown to regulate gene expression through RNA-RNA interactions [34]. To investigate whether *LncPHx2* directly interacts with mRNAs, we adapted a previously described RNA interactome approach [34]. Biotinylated DNA probes targeting *LncPHx2* were used to precipitate endogenous *LncPHx2* and associated RNAs from Hepa1-6 cells. Using this method, we recovered more than 95% of endogenous *LncPHx2* RNAs (Fig 5A). The associated RNAs were sequenced and 415 unique transcripts were identified, suggesting that *LncPHx2* is associated with multiple RNAs (S3 Table). The sequences of associated RNA fragments were subjected to a *de novo* motif search using MEME (Motif-based sequence analysis tools) [35]. We identified a 21-nucleotide motif that was strongly enriched in *LncPHx2*-interacting RNAs (Fig 5B). Interestingly, this motif is present in tandem in both sense and antisense directions in the *LncPHx2* RNA itself (Fig 5B).

We found that mRNAs encoding MCM2, MCM3 and MCMC7, which were upregulated upon *LncPHx2*-depletion in regenerating livers, were associated with *LncPHx2* in Hepa1-6 cells. The MCM2-7 proteins are key components of the DNA pre-replication complex and are required for initiation of eukaryotic genome replication [41]. Both gain- and loss-of-function analyses demonstrated that MCMs promote cell proliferation [42]. The *LncPHx2* RNA-interacting motif is present in these three Mcm mRNAs with high significance as evaluated by MAST (Motif-based sequence analysis tools)[36] (Fig 5B). With the exception of these mRNAs and those listed in S4 Table, we did not observe significant overlap between the mRNAs that are associated with *LncPHx2* in Hepa1-6 cells and those that are differentially expressed upon *LncPHx2*-depletion during liver regeneration. The systems in which these experiments were performed (an *in vitro* cell line vs. regenerating liver) are very different, and *LncPHx2* probably regulates different sets of genes under different biological conditions.

Since it is technically challenging to pull-down endogenous *LncPHx2* and its associated RNAs in regenerating liver, we evaluated *LncPHx2* RNA-interacting motif enrichment in the differentially expressed genes in *LncPHx2*-depleted regenerating livers *in silico*. MAST reverse motif search were performed on similar numbers of genes from upregulated (top 300 out of 531) and downregulated (291 out of 291) gene categories, as the results of MAST is influenced by numbers of sequences submitted for each query [36]. A hundred sets of 300 randomly sampled genes from unchanged gene category were used as control. We found a significant enrichment of the *LncPHx2* motif in the upregulated transcripts. The overall E-value of upregulated transcripts is significantly lower compared to both downregulated transcripts and unchanged transcripts (Fig 5C). These results suggest that *LncPHx2* could downregulate gene expression during liver regeneration, by directly binding to the mRNAs through the identified *LncPHx2* RNA-interacting motif

## Discussion

LncRNAs are often expressed only in certain tissues or during specific developmental stages [1, 4]. In order to characterize lncRNAs that regulate cell proliferation, we used a mouse PHx model, in which cells are synchronized to proliferate in response to the loss of liver mass. A large number of lncRNAs are differentially expressed over the time course of liver regeneration. One lncRNA, *LncPHx2*, which is highly induced during liver regeneration, was studied in detail. Depletion of *LncPHx2* by ASOs before PHx surgery led to more rapid liver mass recovery, increased cell proliferation, and faster recovery of liver function compared to mice treated with vehicle (Fig 3). Genome-wide gene expression profiling showed that depletion of *LncPHx2* during liver regeneration led to upregulation of genes that promote cell proliferation (Fig 4). Recently, several studies have shown that lncRNAs play important roles in regulating cell proliferation by controlling the expression levels of the cell-cycle regulators [43]. *NcRNA*_*ccnd1*_ and *Gadd7* induced by DNA damage, directly regulate the level of CCND1 and CDK6, and contribute to the cell cycle arrest caused by DNA damage [44, 45]. Our data here showed that *LncPHx2* induced by PHx *negatively* impacts the hepatocyte proliferation during the liver regeneration process in response to PHx—a self-tuning mechanism. In eukaryotes, many proteins such as INK4 proteins, CIP/KIP families, and p21 were known to serve as checkpoints to monitor cell cycle progression [46]. Different from these coding negative cell-cycle regulators that can halt cell-cycle progression completely, *LncPHx2* seems to play a moderate role in tuning cell proliferation rate when a robust cell proliferation is induced by PHx surgery. A recent study by Kambara et al showed that an IFN induced *lncRNA-CMPK2* acts as a negative regulator of a subset of IFN-induced gene to prevent excessive or uncontrolled IFN response [47]. Together, long non-coding RNAs add another layer of regulation to achieve the precise control of biological processes.

Interestingly, *LncPHx2* plays a more important role in regulating hepatocyte proliferation in regenerating livers than in normal livers or in liver cell lines. No measurable phenotype was observed in the normal mouse liver when *LncPHx2* was depleted by two different *LncPHx2* ASOs (data not shown). We only observed a slight inhibition of cell proliferation in cell lines when we overexpressed *LncPHx2* to more than 100-fold compared to endogenous level, and no measurable phenotypic changes were observed upon *LncPHx2* depletion (data not shown). Our study provided evidence that lncRNAs function as key modulators under stress conditions [1, 4]. Similarly, LncRNA PRINS (Psoriasis susceptibility-related RNA Gene Induced by Stress) was previously reported to be induced by serum-starvation, and promote cell viability only after serum starvation, but not under normal serum condition [48].

Although GSEA analysis showed that the gene expression profile upon *LncPHx2* depletion in regenerating mouse livers is similar to the gene expression pattern of mouse HCC, and the depletion of *LncPHx2* promoted hepatocyte proliferation after PHx, the depletion of *LncPHx2* did not promote tumorigenesis in the DEN-induced mouse HCC model (S5 Fig). These data suggested that *LncPHx2* is a negative cell proliferation regulator but not a tumour suppressor. However, it is possible that *LncPHx2* only protects against tumour initiation but not progression, which needs to be further investigated.

We searched for a human orthologue of *LncPHx2* and identified a region with sequence homology (Human hg19 chr2:208564953–208576096, 65.8%, UCSC TransMap analysis) in the human genome. We found no evidence of transcriptome activity in this locus in any available database. Since *LncPHx2* is expressed at very low levels in mouse tissues, with the exception of testis, it is possible that expression of the human counterpart of *LncPHx2* is induced under specific conditions, for example in injured livers.

Gene expression profiles showed that the depletion of *LncPHx2* leads to upregulation of cell cycle genes including those encoding E2F1, MCMs, histone proteins, and DNA polymerases (Fig 4). We also showed that in cell lines, *LncPHx2* is localized in the cytoplasm and associates with many mRNAs including *Mcm2*, *Mcm3*, and *Mcm7* (Figs 2 and 5). It is possible that *LncPHx2* downregulates gene expression by binding to these mRNAs and affecting their stability. We also evaluated proteins that are associated with *LncPHx2* using conditions modified to favour RNA-protein binding [49]. The proteins identified were mainly ribosomal subunits (data not shown). There have been reports that lncRNAs associate with ribosomes and generate small peptides [50]. *LncPHx2* contains one open reading frame encoding a peptide with 27 amino acids. We believe it is unlikely that *LncPHx2* is translated. First, the open reading frame in *LncPHx2* is not conserved. Second, the *LncPHx2* transcript lacks a Kozak sequence. Third, no matches were found in mouse or human proteomic databases to date (http://world-2dpage.expasy.org/repository/; http://reprod.njmu.edu.cn/cgi-bin/2d/2d.cgi). Recently, it was reported that lncRNA *GAS5* regulates *c-MYC* translation by association with the ribosome [51]. It is possible that *LncPHx2* regulates both mRNA stability and translation of its associated mRNAs. The detailed mechanism of action of *LncPHx2* remains to be elucidated.

## Supporting Information

S1 Fig Characterization of LncPHx2. (A) qPCR analysis of *Ccnyl1* mRNA expression during liver regeneration; *Ccnyl1* neighbours the *LncPHx2* gene. The RNA levels in the livers of mice subjected to sham surgery at time 0 after surgery were set as 1. Data were quantified and statistically analysed as in Fig 1C. (B) Reported transcription isoforms of *LncPHx2* (Ensembl). (C) qPCR analysis of *LncPHx2* levels in nuclear and cytoplasmic RNA fractions from MHT cells. *LncPHx2* level in total cellular RNA was set as 1.
(EPS)

S2 Fig *LncPHx2* depletion in MHT and BNL.CL2 cells by *LncPHx2* specific ASOs. qPCR analysis of *LncPHx2* levels after ASO treatment of (A) MHT cells and (B) BNL.CL2 cells. The *LncPHx2* levels in untreated (NT) cells were set as 1. Data were quantified and statistically analysed as in Fig 1C.
(EPS)

S3 Fig Depletion of *LncPHx2* leads to faster liver weight recovery at 48 hours after PHx. (A) Experimental procedure. Mice were given two doses of PBS or ASOs at 50 mg/kg (mpk) at time points (indicated by green arrows) before PHx surgery (indicated by red arrow). Animals were sacrificed to collect samples for phenotype study at 48 hours after PHx (indicated by blue arrow). n = 3. (B) qPCR analysis of *LncPHx2* depletion by ASOs in mouse livers 48 hours after PHx. The RNA level in the PBS-treated mouse liver at time 0 after PHx was set as 1. Data were quantified and statistically analysed as in Fig 1C. (C) Liver to body weight ratios were measured at 48 hours after PHx.
(EPS)

S4 Fig Genes promote cell proliferation are enriched in *LncPHx2*-depleted regenerating livers. Top positively associated pathways by GSEA analysis. (A) Enrichment plot, DNA strand elongation (Reactome) (M19312). (B) Enrichment plot, Meiosis (Reactome) (M529). (C) Enrichment plot, Meitotic recombination (Reactome) (M1011). (D) Enrichment plot, Telomere maintenance (Reactome) (M4052).
(EPS)

S5 Fig *LncPHx2* depletion does not promote tumour progression in DEN-induced HCC mouse model. (A) Enrichment plot of top negatively associated pathways by GSEA analysis. Left panel: Liver cancer DENA downregulated (Lee) (M1424): top 100 downregulated genes in mouse DEN-induced HCC. Right panel: Liver cancer MYC E2F1 downregulated (Lee) (M5636): top 100 downregulated in HCC from Myc/E2f1 double transgenic mice. (B) Enrichment plot of top positively associated oncogenic pathways by GSEA analysis. RB1 and RBL1 KO upregulated (M2802): top 150 upregulated genes in primary keratinocytes from RB1 and RBL1 skin specific knockout mice. BRCA1 KD upregulated (M2748): top 150 upregulated genes in MCF10A cells upon knockdown of BRCA1 gene by RNAi. (C) qPCR analysis of *LncPHx2* levels in DEN-induced mouse HCC tumour. The RNA level in the adjacent normal tissue was set as 1. Data were quantified and statistically analysed as in Fig 1C. N = 8. (D) Schematic of DEN-HCC model study design. (E) qPCR analysis of *LncPHx2* levels in tumour and adjacent normal tissues from mice treated with LncPHx2_ASO1 or a control ASO. *LncPHx2* levels in adjacent normal tissues of mice treated with PBS were set as 1. Data were quantified and statistically analysed as in Fig 1c. N = 10–12. (F) Numbers of tumours in mice treated as indicated. N = 10–12
(EPS)

S1 Table Differentially expressed mRNAs and lncRNAs during liver regeneration after PHx. (XLSX)

S2 Table Differentially expressed genes at 48 hours after PHx and sham surgery in PBS- and LncPHx2_ASO1-treated mouse livers. (XLSX)

S3 Table Transcripts identified in *LncPHx2* RNA-interactome study. (XLSX)

S4 Table Transcripts identified in both RNA-seq analyses in livers treated with LncPHx2_ASO1 and *LncPHx2* RNA-interactome study. (XLSX)
